# 3D multicellular systems in disease modelling: From organoids to organ-on-chip

**DOI:** 10.3389/fcell.2023.1083175

**Published:** 2023-02-02

**Authors:** Caoimhe Goldrick, Ina Guri, Gabriel Herrera-Oropeza, Charlotte O’Brien-Gore, Errin Roy, Maja Wojtynska, Francesca M. Spagnoli

**Affiliations:** Faculty of Life Sciences, Centre for Gene Therapy and Regenerative Medicine, Guy’s Campus, King’s College London, London, United Kingdom

**Keywords:** organoids, assembloids, organ-on-chip, multicellular systems, disease modelling

## Abstract

Cell-cell interactions underlay organ formation and function during homeostasis. Changes in communication between cells and their surrounding microenvironment are a feature of numerous human diseases, including metabolic disease and neurological disorders. In the past decade, cross-disciplinary research has been conducted to engineer novel synthetic multicellular organ systems in 3D, including organoids, assembloids, and organ-on-chip models. These model systems, composed of distinct cell types, satisfy the need for a better understanding of complex biological interactions and mechanisms underpinning diseases. In this review, we discuss the emerging field of building 3D multicellular systems and their application for modelling the cellular interactions at play in diseases. We report recent experimental and computational approaches for capturing cell-cell interactions as well as progress in bioengineering approaches for recapitulating these complexities *ex vivo*. Finally, we explore the value of developing such multicellular systems for modelling metabolic, intestinal, and neurological disorders as major examples of multisystemic diseases, we discuss the advantages and disadvantages of the different approaches and provide some recommendations for further advancing the field.

## 1 Introduction

Cell–cell interactions orchestrate numerous cellular processes during embryonic development as well as adult tissue homeostasis and their alterations can lead to disease. Thus, a full understanding of cellular interactions is critical to many physiological and pathological processes. However, this requires appropriate experimental models to faithfully reproduce the *in vivo* cellular microenvironment. To date, two-dimensional (2D) culture systems have mostly been employed to investigate cellular interactions (Duval et al., 2017). These models rely on the adherence of cells to flat culture plates, offering mechanical support and access to nutrients from the culture media. 2D models can be useful for a preliminary investigation to assess the direct effects of 1 cell type on another, however, their simplistic structure deviates substantially from the highly complex *in vivo* microenvironment which consists of an array of cell types, chemical gradients, soluble factors, mechanical environment (matrix and interstitial flow), and gases (oxygen, carbon dioxide) (Huh et al., 2011).

**FIGURE 1:**
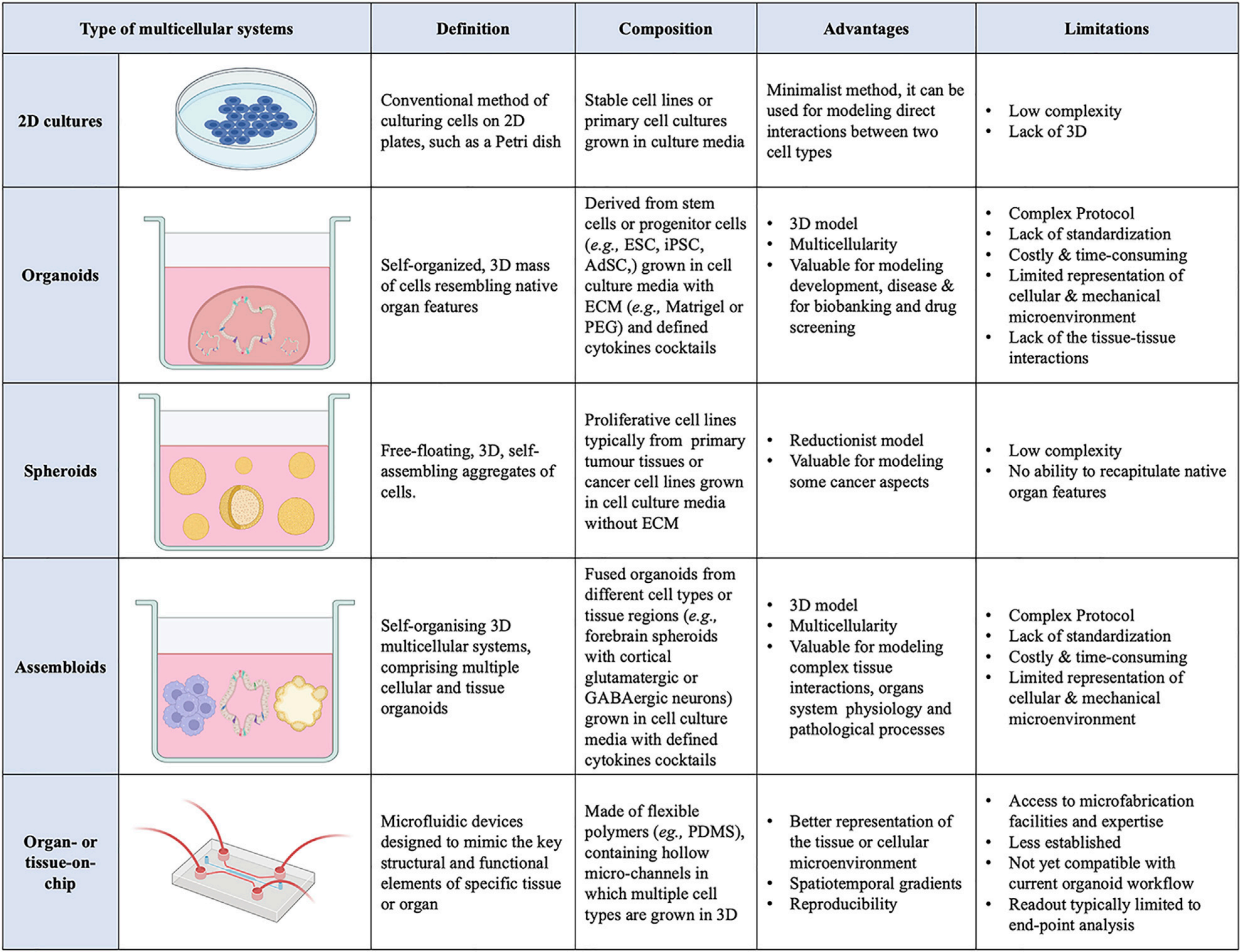
List of current 3D multicellular systems, showing how they are engineered, and the advantages and disadvantages of each system.

In the last decade, cross-disciplinary research has enabled advances in modelling cellular interactions through the generation of synthetic three-dimensional (3D) multicellular systems (Huh et al., 2011; Knight and Przyborski, 2015). These 3D model systems better recapitulate the *in vivo* biochemical and biomechanical microenvironment (Pampaloni et al., 2007; Huh et al., 2011) and have rapidly superseded 2D culture methods. 3D multicellular systems use culture techniques, such as growing cells in an extracellular matrix (ECM), which improve cell differentiation, growth, migration, and tissue organisation compared to 2D models (Duval et al., 2017). A large variety of 3D multicellular systems has emerged in the last decade, including organoids and spheroids, and more recently assembloids and organ- or tissue-on-chip systems (Figure 1).

**FIGURE 2:**
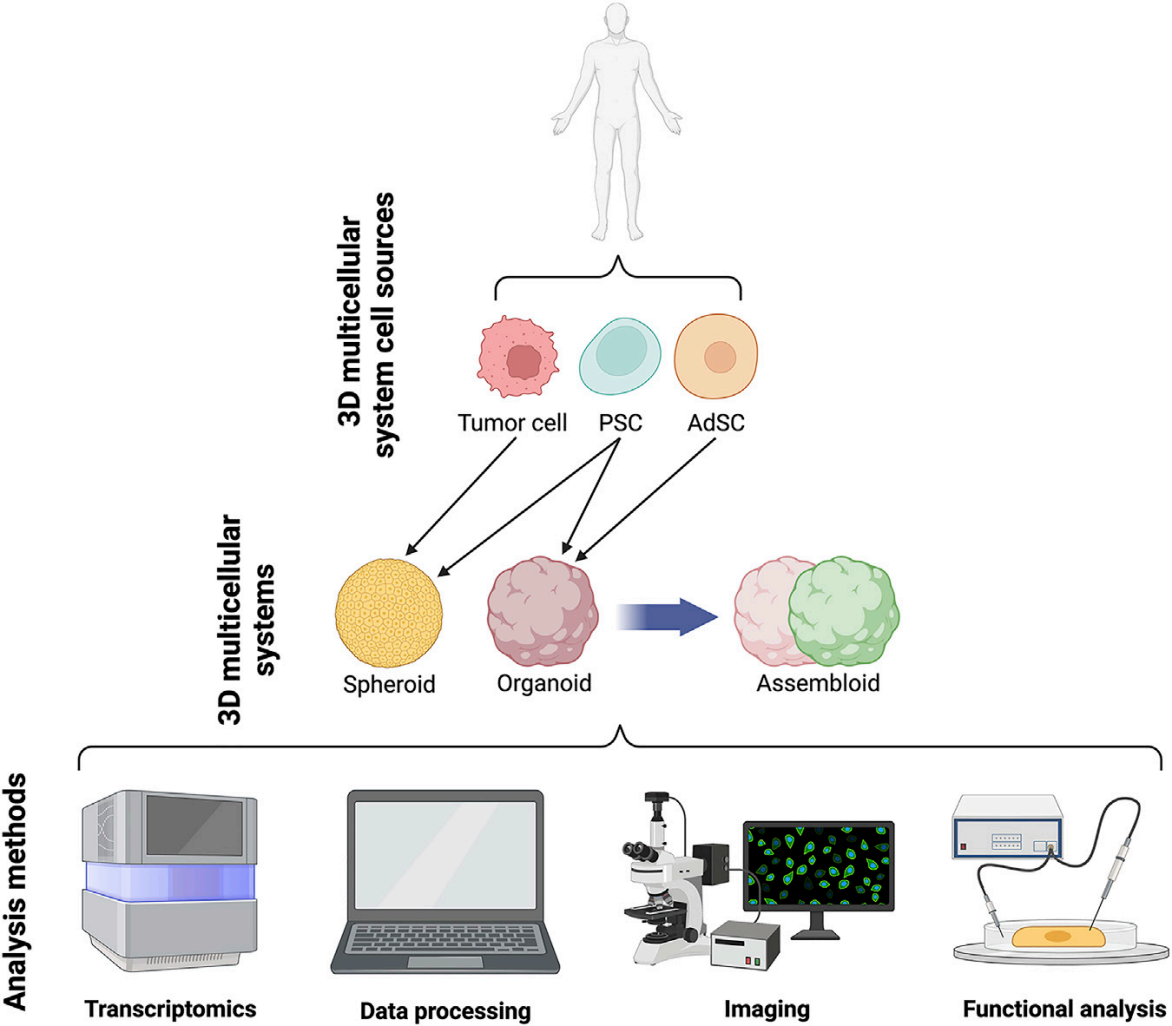
Schematics of current 3D systems from cell lines to spheroids, organoids and assembloids and downstream analyses.

Organoids are self-organising 3D cell aggregates capable of recapitulating the cellular heterogeneity, *in vivo*-like features, structure, and functions, to a certain extent, of human organs. Crucially, organoids are grown in an ECM which provides mechanical support to the cells. Organoids can be derived from various sources of mouse or human stem cells, including embryonic stem cells (ESCs), adult tissue-resident stem cells (AdSCs) and induced pluripotent stem cells (iPSCs) (Camp et al., 2015; Knight and Przyborski, 2015; de Jongh et al., 2022; Morais et al., 2022). Hence, human organoids represent a powerful 3D multicellular system for modelling human-specific aspects of development and disease, bridging the gap between 2D cell culture methods and animal models (Figure 1 and Figure 2). Seminal work in 2008 from Sasai and others reported the first evidence of stem cell-derived neural cell 3D cultures that self-organized into cortical-like structures, laying the foundations for brain organoid protocols (Eiraku et al., 2008). Concomitantly, Clevers and co-workers were the first group to engineer intestinal organoids from AdSCs, work that widely influenced the future development of multicellular systems (Sato et al., 2009). Since, the field has grown rapidly and protocols for the generation of different types of organoids have been developed, such as lung (Dye et al., 2015), gastric (McCracken et al., 2014), retina (Eiraku et al., 2011), kidney (Takasato et al., 2015), liver (Takebe et al., 2013) and brain (Lancaster et al., 2013) organoids. These model systems can be used to study a range of biomedical, developmental, and disease-related questions.

A 3D multicellular system similar to organoids are the spheroids. The nomenclature surrounding spheroids and organoids can be ambiguous, with the terms sometimes used interchangeably. Spheroids are 3D free-floating, self-assembling aggregates of cells that are generated without the use of a scaffold matrix. Scaffold-free spheroid assembly can be achieved using several techniques including pellet culture, liquid overlay, hanging drop, spinner culture, rotating wall vessel, microfluidics, and magnetic levitation (Ryu et al., 2019; Białkowska et al., 2020). The main difference between spheroids and organoids is the nature of their assembly, with spheroids being free-floating whilst organoids are maintained within an ECM scaffold. As such, spheroids are generally regarded as more reductionist models compared to their organoid counterparts, mainly due to the non-use of scaffolding matrix, making spheroids a less stable and less complex structure (Gunti et al., 2021). Spheroids can be generated from any proliferative cell type, which possesses the ability to self-aggregate, but are typically derived from cancer cell lines and dissociated tumor cells (Velasco et al., 2020). An advantage of the spheroid tumor models is their ability to preserve to some extent the heterogeneity observed in patients’ primary tumors. Moreover, the ease of spheroids generation makes them amenable to high-throughput protocols and popular for drug screening (Białkowska et al., 2020). By contrast, organoid models often require lengthier, complex protocols, with the pay-off of capturing more diverse organ-specific cell types that are highly spatially organized.

A more recent advance in 3D multicellular systems has been the development of assembloids (Figure 1 and Figure 2). First coined by Sergiu Paşca (Vogt, 2021), assembloids are the next generation of 3D multicellular systems, combining together multiple brain region–specific organoids and/or other lineages’ organoids or spheroids (Birey et al., 2017; Kanton and Paşca, 2022). Assembloids have proven to be valuable models for investigating cell-cell interactions, as they integrate multiple cellular systems. They are particularly useful to capture interactions between different brain regions, allowing to study the assembly of neural circuit and to model neurological diseases (Schmidt, 2021). Paşca’s group was among the first to engineer assembloids by combining forebrain spheroids with cortical glutamatergic or GABAergic neurons to model the saltatory migration of interneurons in the fetal forebrain (Birey et al., 2017). Since then, many assembloid models have been designed to study developmental biology and neuroscience (Makrygianni and Chrousos, 2021; Panoutsopoulos, 2021), including neural circuits (Miura et al., 2022), neuropsychiatric disease (Miura et al., 2020), cortico-motor interactions (Andersen et al., 2020), and neuro-muscular information flows (Koike et al., 2019). Besides cellular interactions within brain organoids, assembloid-like approaches modelling different regions of the gastrointestinal (gi) tract, from foregut to hindgut (Koike et al., 2019), as well as the endometrium (Rawlings et al., 2021) have started to emerge. Thus, the use of assembloids is gaining increasing popularity, owing to the level of complex interactions they can unveil, which in turn can increase our understanding of organ physiology and pathological processes (Kanton and Paşca, 2022). Assembloids of the future may even assemble organoids from different systems, enabling the study of multisystemic diseases, such as autoimmune encephalitis (Marton and Pașca, 2020; Makrygianni and Chrousos, 2021) where the immune system comes into contact with the nervous system.

In this review, we discuss the relevance of 3D multicellular systems in biomedical research, encompassing approaches for generating different types of 3D multicellular systems and their implementation in disease modelling. Many excellent reviews have been recently published on 3D multicellular systems in modelling cancer, infectious diseases, and genetic disorders (Drost and Clevers, 2018; Perez-Lanzon et al., 2018; Kim et al., 2020; Shpichka et al., 2022). Here, we focus instead on recent applications of organoids and assembloids in modelling gi, metabolic, and neural disorders as major examples of multisystemic diseases. Finally, we provide recommendations for moving the field forward.

## 2 Engineering 3D multicellular systems

Organoids rely on the self-organization properties of stem cells. Typically, to generate organoids, pluripotent stem cells are first subjected to directed differentiation by exposure to a combination of inductive signals, which recapitulate physiological cues found during embryonic development, and then aggregated in 3D structures, often embedded into ECM matrixes (Velasco et al., 2020; Corsini and Knoblich, 2022). By contrast, in AdSC-derived organoids a faithful *in vitro* re-creation of their stem cell niche sustains self-renewal, allowing concomitant differentiation and self-organization into 3D structures (Sato et al., 2009).

Efforts in the field have concentrated on exploring the principles underpinning self-organization but also on designing biomaterials and biofabrication methods to guide and improve the self-organization processes (Hofer and Lutolf, 2021). Indeed, the introduction of some external constraints might reduce the high variability of the organoid system and enhance their translatability and physiological relevance (Brassard et al., 2021). For instance, self-organization might be somehow constrained through spatiotemporal control of cell-cell and cell-ECM interactions that are at play in the stem cell niche(s). Tackling this problem will require exchanges and concerted efforts from computational scientists, stem cell biologists and bioengineering experts to develop the right approaches.

### 2.1 Engineering approaches for 3D multicellular systems

**FIGURE 3:**
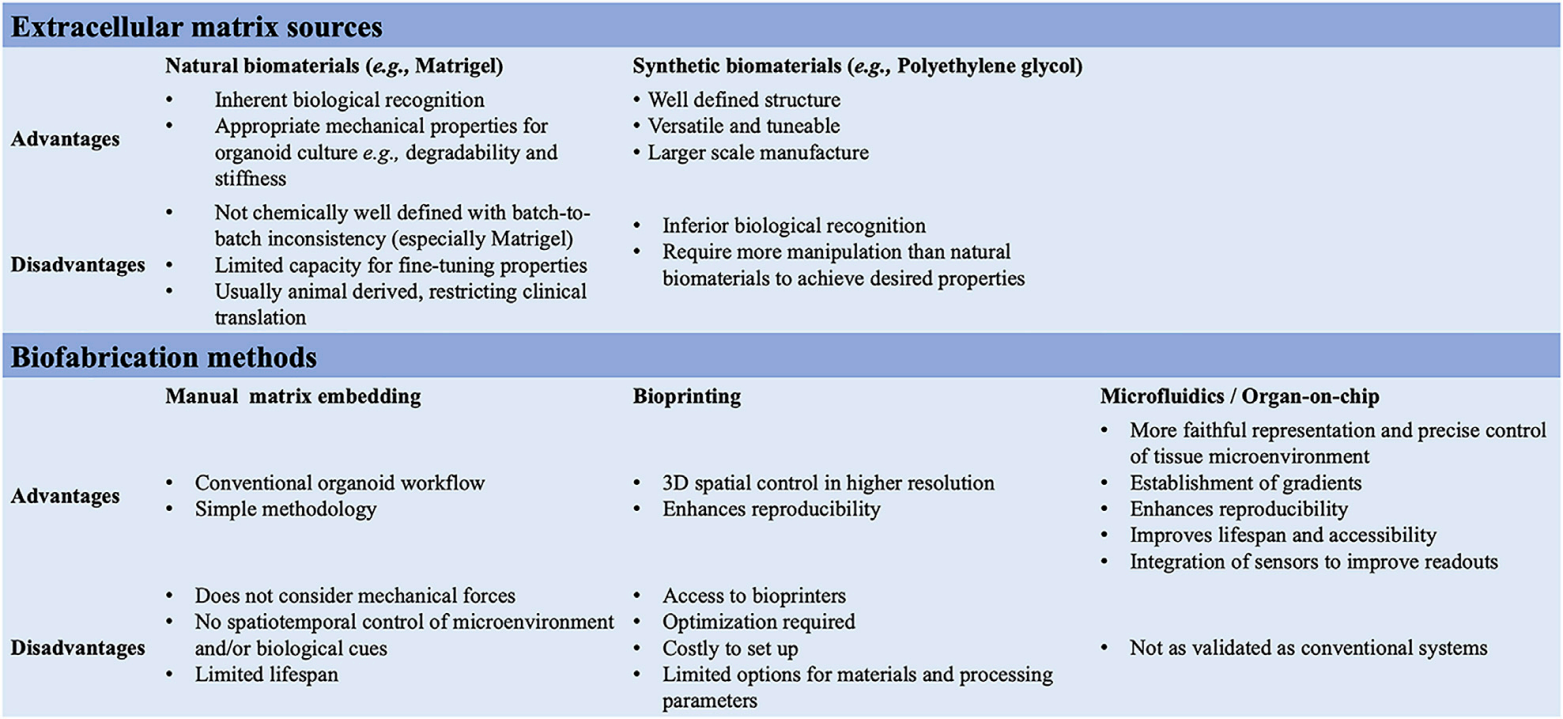
Summary of the ECM sources and biofabrication methods for the generation of 3D multicellular systems.

In 3D organoid cultures, hydrogels are among the most used biological scaffolds, providing mechanical support for cells, creating artificial cellular microenvironment(s), and promoting cell–cell and cell-ECM interactions (Li et al., 1987; Sato et al., 2009). Hydrogels are cross-linked networks of water-swollen hydrophilic polymers either of natural origin formed from biologically derived precursors, such as ECM proteins, or synthetic derived from non-natural molecules (Kleinman and Martin, 2005; Jabaji et al., 2014; Broguiere et al., 2018) (Figure 3). Matrigel, a laminin-rich artificial ECM, is the best-known hydrogel used in organoid cultures. While Matrigel provides a complex ECM signalling network and has suitable mechanical properties for organoid cultures, its composition is not well defined, with batch-to-batch inconsistency, introducing additional variability and hampering reproducibility. Additional limitations of Matrigel are the limited capacity for customization and tuning and its animal-derived origin, from a mouse sarcoma, which restricts its clinical translation (Aisenbrey and Murphy, 2020; Magno et al., 2020; Hofer and Lutolf, 2021; Kozlowski et al., 2021). Synthetic hydrogels might represent a valuable alternative for organoid cultures; they are chemically well-defined, offer superior control over the material properties, and can be manufactured on a larger scale (Lutolf and Hubbell, 2005; Gjorevski et al., 2016; Ranga et al., 2016; Aisenbrey and Murphy, 2020). For instance, synthetic biomaterials allow the modulation of individual parameters (*e.g.,* stiffness, viscoelasticity, degradation) on cell behaviour, in a reductionist and stepwise manner (Hofer and Lutolf, 2021; Lust et al., 2021). The most common synthetic biomaterial that has been used for organoid cultures is polyethylene glycol (PEG), owing to its high tunability, design flexibility, resistance to absorption, and low cost (Caliari and Burdick, 2016; Foyt et al., 2018; Kratochvil et al., 2019). PEG-based hydrogels are inherently inert but can be functionalized through the addition of adhesive peptides, including RGD and IKVAV motifs for fibronectin and laminin, respectively (Foyt et al., 2018). These integrin-binding domains can influence intercellular communication depending on their concentration, spacing, presentation timing, and patterning (Kratochvil et al., 2019). PEG-based hydrogels can also be cross-linked with degradable crosslinkers (Caliari and Burdick, 2016). Indeed, cells *in vivo,* in their native microenvironment, constantly remodel their surroundings by degrading the ECM and synthesizing nascent proteins. Thus, by controlling the degradation of PEG hydrogels, one can fine-tune the biomaterial to mimic the *in vivo* microenvironment remodelling and its influences on cell behaviour and intercellular communication (Kratochvil et al., 2019). For example, proteolytically ADAM9-degradable 3D hydrogel systems have been recently reported to enable neural stem cells spreading and to facilitate cadherin-mediated cell-cell contact along with downstream β-catenin signalling, which in turn maintains their stemness (Halbleib and Nelson, 2006; Madl et al., 2017). This suggests that matrix remodelling might be a generalizable strategy for stemness maintenance in 3D. Controlled degradation may also offer spatiotemporal control onto the release of specific biochemical cues that can be regulated to guide the differentiation of embedded stem cells (Kloxin et al., 2009; Wylie et al., 2011). The PEG-4MAL is another class of PEG-based hydrogels with an additional chemical modification, which has demonstrated high versatility for the on-demand delivery of growth factors and recombinant proteins in various tissue regeneration applications (Weaver et al., 2017). For instance, human intestinal organoids have been successfully generated following encapsulation in PEG-4MAL hydrogels functionalized with adhesive peptide and degradable linker (Cruz-Acuña et al., 2018). This hydrogel system not only supported the organoid growth but also enhanced their engraftment and colonic wound repair in a mouse model of intestinal injury, presenting an attractive alternative to Matrigel (Cruz-Acuña et al., 2018).

Additionally, organoids assembly approaches have started to emerge with the aim to achieve cellular complexity in a controlled manner and inspired by organ development (Takebe and Wells, 2019). Wells and others have pioneered the field, generating multicellular gastric organoids by combining cells from distinct germ layers (enteric neuroglial, mesenchymal, and gastric epithelial precursors) that were derived separately from human pluripotent stem cells and then mixed together (Eicher et al., 2022) (see more examples discussed below in different sections). Novel biofabrication technologies are needed to combine these different cell types in appropriate ratios and spatial patterns as *in vivo* in the tissues and control the organization of 3D multicellular systems. Bioprinting is one such technology that uses bioinks, biomaterials containing live cells, to control the geometry in 3D (Barreiro Carpio et al., 2021). This can facilitate the self-organization of multicellular systems through spatiotemporal control of cell-cell and cell-ECM interactions with higher reproducibility, resolution, and throughput (Brassard et al., 2021; Lawlor et al., 2021). However, there is presently a limited scope for bioprinting organoids as meeting all the necessary equipment, material, chemical and bioprocessing constraints is challenging (Figure 1).

Beyond local cell-cell and cell-ECM interactions, cells *in vivo* undergo mechanotransduction in response to external stimuli, such as mechanical forces, flow, and pressure (Shi and Tarbell, 2011). These forces influence cell behaviour and should be considered in the engineering design efforts of mature organoids. Additionally, static 3D organoids rely on passive diffusion for nutrient transfer and waste removal, which becomes insufficient with increasing size, again hindering organoid maturation (Park et al., 2019). The organ-on-chip technology may offer the integration of such stimuli into organoid cultures (Huh et al., 2010; Ingber, 2022). Organ-on-chip models are microfluidic devices that are designed to mimic the key structural and functional elements of a specific tissue or organ (Figure 1). These models are typically made of transparent, flexible polymers, such as polydimethylsiloxane (PDMS), containing hollow micro-channels in which multiple cell types are grown in 3D. They often incorporate physiological biomechanical forces, such as flow, to simulate the microenvironment of the tissue or organ being studied, so that cells interact in a manner that closely resembles their native environment (Low et al., 2021). The controlled environment of organ-on-chip models allows researchers to study complex cell-cell interactions more easily, investigate relevant signalling pathways, gain insight into disease mechanisms and study the effects of different drug treatments (Ewart et al., 2022). Organ-on-chip devices can also be set up in a way that supports co-culture systems, by connecting chambers of different organ-on-chips (Zhang et al., 2017). This has opened possibilities for engineering vascularized and multi-organ systems to model tissue-tissue or even multi-organ interactions to investigate more systemic interactions, rather than just those within one single organ (Bauer et al., 2017; Jin et al., 2018; Homan et al., 2019; Hofer and Lutolf, 2021). Organoids and organ-on-chip devices have started as distinct approaches, though now scientists are beginning to explore combining them to utilize the benefits of both (Park et al., 2019). For example, kidney organoids cultured in chips, once subjected to physiological flow rates to simulate fluidic shear stress, displayed enhanced maturity compared with static controls (Homan et al., 2019). Similarly, an intestinal organoid-on-chip was recently developed with an *in vivo*-mimicking spatial arrangement and an accessible and perfusable lumen, allowing for the continuous removal of waste products to increase lifespan. Moreover, in this organoid-on-chip model rare, specialized cell types of the gut were identified that were not found in conventional control intestinal organoids (Nikolaev et al., 2020).

When organoid-on-chip models are combined with microfluidics, morphogen gradients can be introduced to induce extrinsic controlled symmetry breaking of stem cell colonies, aiding self-organization for intestinal organoid development (Manfrin et al., 2019; Hofer and Lutolf, 2021). Similarly, very recent technology has been developed to establish gradient culture platforms using a scaffold-based localization approach, referred to as Gradient-in-CUBE workflow (Koh and Hagiwara, 2022). The CUBE culture device is modular and enables a localized supply of morphogens as well as ECM-protein solutions to spheroids/organoids undergoing differentiation, without the need for a microfluidic setup (Koh and Hagiwara, 2022). This type of approach may help devise future strategies to generate increasingly complex multilineages and regionally patterned organoid structures, such as assembloids. In summary, recent observations suggest that guiding the self-organization properties of organoids by applying engineering principles is the path to success toward the generation of more physiologically relevant *in vitro* multicellular models. Coupling organoids with bioengineering technology including organs-on-chip to model *in vivo* multicellular systems may open new frontiers in regenerative medicine.

### 2.2 Characterization of engineered 3D multicellular systems

3D multicellular systems are routinely analysed using various methods, including image-based analysis, biochemical, molecular, and functional characterization (Hofer and Lutolf, 2021; Keshara et al., 2022). A thorough characterization of the established organoids or any 3D systems is paramount and requires systematic benchmarking against primary tissue to fully assess the extent to which the models actually recapitulate endogenous cellular composition, tissue architecture and functionalities.

Several innovative imaging-based strategies have been developed and are routinely applied to analyze and quantify features of organoids grown *in vitro*, including confocal microscopy, light-sheet microscopy, and live-imaging (for detailed reviews please refer to (Hofer and Lutolf, 2021; Keshara et al., 2022). Single-cell RNA-sequencing (scRNA-seq) has also rapidly become a valuable approach to characterize the composition of organoids grown *in vitro,* allowing direct comparison against their primary tissues through datasets integration (Camp et al., 2015; Haber et al., 2017; Sloan et al., 2017; Xiang et al., 2017). Notably, single-cell transcriptomics approach has enabled to unveil cellular heterogeneity and detect the presence of rare cell types in intestinal organoids (Grün et al., 2015; Haber et al., 2017; Beumer et al., 2018). Additionally, applying scRNA-seq on 3D models has started to shed light on cell-cell interactions and their role(s) during human organ development, which is relatively unexplored for ethical and practical reasons. To date numerous, bioinformatic methods have been developed to infer cell-cell interactions from scRNA-seq datasets, including CellPhoneDB, NicheNet, iTalk, CellTalker, CellChat (Wang et al., 2019; Browaeys et al., 2020; Cillo et al., 2020; Efremova et al., 2020; Gonçalves et al., 2021). For instance, the InterCom computational tool was used to generate a map of intercellular communications between human pancreatic progenitors and surrounding microenvironment and the information was then used for improving the 3D culture conditions for pancreatic progenitor expansion (Gonçalves et al., 2021). Similarly, other groups took advantage of scRNA-seq and computational analyses combined with the modular nature of the organoids to systematically screen cellular interactions responsible for inducing growth and/or differentiation in different human tissues (Holloway et al., 2020; Garcia-Alonso et al., 2021). The next step is to apply such knowledge to further increase the complexity of 3D multicellular systems with the aim to mimic the native tissues more accurately and to engineer more reproducible structures, which in turn can serve as better models.

Although cell-cell interactions can be computationally predicted, scRNA-seq datasets do not preserve spatial information, and so these predictions still require extensive experimental *in situ* validation in the tissue. Spatial transcriptomics methods are emerging as promising strategies as they bridge the gap between measuring gene expression and spatial analysis of where cells sit within a tissue while preserving the spatial location of each expression datapoint in a tissue sample (Walker et al., 2022). It is very likely that scRNA-seq and spatial transcriptomics will be applied more and more in combination for high-throughput analyses of organoid models. This framework will be crucial not only for developmental studies but also in the context of disease modelling as it allows to define the specific roles of individual cell types in diseases (Bershteyn et al., 2017; Haber et al., 2017). Finally, as compared to the rapid development of imaging and transcriptomics approaches, assays for a proper functional characterization of the organoids lag behind (Hofer and Lutolf, 2021). Future works should focus on filling this gap, establishing better and more accessible readouts for direct evidence about the functional state of cells.

## 3 3D multicellular systems in modelling diseases

### 3.1 3D multicellular systems in gastrointestinal and metabolic disease modelling

3D multicellular systems are increasingly used to model the crosstalk between different cell types in the human gi tract. The mammalian gi tract is indeed a highly complex organ system comprising a heterogeneous population of cells, including immune, neural, and epithelial type cells (Takebe and Wells, 2019; Eicher et al., 2022). Therefore, models, which have the capacity to incorporate many different cell types, can better capture the complexity of cell–cell interactions underpinning gi tract development, adult homeostasis, and diseases. Pioneering work from the Wells lab. established stem cells-derived human intestinal organoids, which contained functional enterocytes, as well as goblet, Paneth and enteroendocrine cells, for studying not only human intestinal development but also congenital gut disorders (Spence et al., 2011). Subsequently, they expanded such model by developing an assembly approach to incorporate cell types from all three primary germ layers into the organoids, including gi epithelial, enteric neuroglial and mesenchymal precursors; all 3 cell types were derived from human pluripotent stem cells (Workman et al., 2017; Eicher et al., 2022). This multicellular 3D system opened up opportunities for studying non-cell autonomous mechanisms driving gi development and diseases, such as the Hirschsprung’s disease, characterised by the congenital lack of enteric nerves (Workman et al., 2017). Notably, pluripotent stem cells harbouring Hirschsprung’s-disease-causing mutations in *PHOX2B* gene failed to differentiate into neural crest and enteric neural progenitor cells, which in turn affected the maturation of smooth muscles in the 3D multicellular model, recapitulating the disease phenotype (Workman et al., 2017).

Intestinal organoid cultures have also emerged as a powerful platform to model intestinal diseases caused by chronic inflammation or physical injury, including inflammatory bowel disease (IBD), short bowel syndrome, coeliac disease, and cystic fibrosis (Fair et al., 2018). IBD is a complex multifactorial disease, due to dysregulated innate and adaptive immune responses against antigens present in the gi tract (de Souza and Fiocchi, 2016). Studying IBD in a 3D multicellular model has unveiled the critical role of the intestinal epithelial cells in the disease and allowed to decipher *in vitro* the multicellular maintenance of gut barrier integrity (Okamoto and Watanabe, 2016). In a study by Noel et al. (2017) the co-culturing of intestinal enteroids with human M0 macrophages demonstrated the role of the macrophages in improving barrier function and maturity of intestinal cells. Here, primary human cells were used to recapitulate the *in vivo* interactions of innate immune cells with the intestinal epithelium. A further advancement of this multicellular system could be to use it for exploring the roles of effector M1 and M2 macrophages, which are important in IBD etiology. Similarly, Bimczok et al. (2015); Daou et al. (2021) employed primary human cells in their multicellular gi to assess the involvement of the small intestinal mucosa in the immune response to chronic gastritis. Specifically, by co-culturing primary gastric epithelial cells with monocytes, they unveiled the secretory potential of epithelial cells in releasing retinoic acid for regulating gastric immune response. In the context of gi disorders with a genetic component, patient-derived intestinal organoids offer the possibility to study the genetic susceptibility or better decipher the underlying molecular mechanisms, representing a better suited model compared to stem cell derived organoids (Fair et al., 2018). Moreover, intestinal organoids generated from patients suffering from coeliac disease or cystic fibrosis have been used as a drug-screening platform for the identification of novel therapies (Dekkers et al., 2013).

3D multicellular systems have proven particularly useful in modelling host-microbiome interactions in intestinal health and disease. In fact, this approach has helped to identify new roles for aerobic and anaerobic gut microbiota and, possibly, the future development of microbiome-related therapeutics and probiotics. Specifically, multicellular models enabled studying human intestinal epithelia in co-culture not only with aerobic but also anaerobic microbiome components, which are the most abundant bacterial species within the human gut (Jalili-Firoozinezhad et al., 2019). Extended cocultures were established using microfluidic intestine-on-a-chip which accurately reconstituted oxygen concentrations and gradients (Jalili-Firoozinezhad et al., 2019). Similar microfluidic devices could also be used to recapitulate other important features of the *in vivo* intestine, such as barrier function and immune components (Gijzen et al., 2020).

Furthermore, the use of 3D multicellular systems has great potential to shead light into metabolic diseases affecting the liver (Pournasr and Duncan, 2017; McCarron et al., 2019; Ouchi et al., 2019; Rogal et al., 2019; Duan et al., 2020; Lee-Montiel et al., 2020; Ramli et al., 2020; Yang et al., 2020; Daou et al., 2021; Freag et al., 2021; Slaughter et al., 2021), as well as diabetes mellitus (Xu et al., 2008; Iori et al., 2012; Nguyen et al., 2017; Nikolaev et al., 2020; Tao et al., 2022) and obesity (Viravaidya and Shuler, 2008; Xu et al., 2008; Lee et al., 2019; McCarthy et al., 2020). Metabolic diseases indeed affect a broad range of cell types and organs, with patients often displaying comorbidities, and can be connected to dysbiosis of the gut microbiota (Hu and Lazar, 2022). Thus, the use of 3D multicellular systems is highly beneficial for modelling this group of diseases. For instance, 3D multicellular liver models have been previously shown to accurately model the cellular phenotypes associated with non-alcoholic steatohepatitis (NASH), a metabolic disease affecting the liver (McCarron et al., 2019; Ouchi et al., 2019; Duan et al., 2020; Lee-Montiel et al., 2020; Ramli et al., 2020; Yang et al., 2020; Daou et al., 2021; Freag et al., 2021; Kostrzewski et al., 2021; Slaughter et al., 2021). Daou et al. (2021) established a liver 3D multicellular system comprising a primary human hepatocyte (PHH) model of lipotoxicity, a primary human macrophage (PHM) model, and a primary human hepatic stellate cell (HSC) model. The individual components of this tri-culture encompassed metabolic, inflammatory, and fibrosis aspects of NASH, respectively. In this model, PHHs were separated from PHMs and HSCs by a synthetic porous membrane in transwell culture plates and recapitulated important features of NASH phenotypes (Daou et al., 2021). Takebe and others established multi-cellular human liver organoid-based steatosis and steatohepatitis models (Ouchi et al., 2019) starting from hepatocytes and stromal lineages, such as stellate- and Kupffer-like cells, all derived from pluripotent stem cells. These multicellular human organoids recapitulated key features of inflammation, typically found in steatosis, as well as associated fibrosis phenotypes (Ouchi et al., 2019). In a more recent study, the same model was used to develop a functional genomics framework to screen for clinically relevant non-alcoholic fatty-liver disease (NAFLD)/NASH phenotype-genotype associations in different metabolic states (Kimura et al., 2022).

In the field of diabetes, a variety of microfluidic devices have been introduced to recreate native islet microenvironments, to mimic pancreatic β-cell kinetics *in vitro* as well as pancreatic β-cells and target tissues (e.g., pancreas-liver) organ-on-chip models were developed to model type 2 diabetes (Jun et al., 2019). Also, microfluidic vascularized micro-organ platforms have been designed to incorporate human cadaveric islets within a 3D vascularized tissue (Bender et al., 2021). Such platforms may mimic important aspects of the native islet niche with β-cells receiving glucose *via* the surrounding vasculature as well as immune cells, reproducing the early stages of T1D disease progression (Bender et al., 2021). Ongoing efforts in the field are directed toward engineering 3D multicellular islet models starting from human pluripotent stem cell-derived pancreatic cells along with supporting cells and vessels for disease modelling and cell therapy (Augsornworawat et al., 2019; Aghazadeh et al., 2021; Glorieux et al., 2022).

### 3.2 3D multicellular systems in neural disease modelling

Human brain organoids are self-organising 3D stem cell cultures that recapitulate many aspects of the early developing human brain. These structures mimic the fetal brain in cellular composition, with progenitor neuronal and glial cell types detected (Lancaster et al., 2013), as well as general tissue structure and developmental trajectory. Prior to the development of this technology, gaining *in vivo* developmental insight was only possible from animal models or fetal tissue. Both entail significant limitations, the former does not fully recapitulate the complexity of the human central nervous system (CNS), while the latter is relatively inaccessible and only available at later developmental time points. Thus, the advent of organoid technology in combination with iPSCs and advanced genome engineering has provided a unique opportunity to model human brain development and function and to study diseases of the nervous system. Cortical organoids were first developed by Lancaster et al. (2013) (15) and have been used in combination with iPSCs to model neurological diseases, such as microcephaly (Omer Javed et al., 2018; Zhang et al., 2019), macrocephaly (Li et al., 2017), lissencephaly (Bershteyn et al., 2017; Iefremova et al., 2017), autism (Mariani et al., 2015), schizophrenia (Ye et al., 2017), down syndrome (Xu et al., 2019), and neuronal heterotopia (Klaus et al., 2019). More recently, Pellegrini et al. (2020) (134) established a more advanced protocol to develop choroid plexus organoids with a selective barrier and cerebrospinal fluid-like secretion. Interestingly, these organoids can predict CNS permeability to new compounds and were used as a model to study SARS-CoV-2 entry through the blood-brain barrier.

Recent years have seen the development of next-generation organoids, such as the assembloids (Figure 1). These models can combine multiple organ regions and/or cell lineages in 3D culture (Schmidt, 2021). They are a particularly valuable tool in neuroscience, as they can be used to model interactions between different brain regions or different CNS/peripheral nervous system components, capture cell–cell interactions, and study the assembly of neural circuits. Patient-derived organoids can be genetically manipulated or infected with pathogens and, subsequently, used in assembloids for studying disease processes in a human context. Several groups have generated multi-region assembloids by fusing together organoids resembling the dorsal and ventral forebrain (Bagley et al., 2017; Birey et al., 2017; Xiang et al., 2017; Sloan et al., 2018; Birey et al., 2022). This model has been used to study the developmental trajectory, neuronal migration, and further interactions between cortical glutamatergic neurons and GABAergic interneurons. Moreover, by deriving forebrain assembloids from patients with Timothy syndrome, a neurodevelopmental disorder caused by mutations in the Ca_V_1.2 calcium channel, Birey et al. (2022) (138) demonstrated a defect in interneurons migration. They also found that acute pharmacological modulation of Ca_V_1.2 can regulate the saltation length, but not the frequency, of interneuron migration in TS (138). This work thus paves the way for neural assembloids to be used as drug-screening platforms in neurological diseases associated with aberrant connectivity.

Assembloid models have also been established for modelling brain connectivity to various CNS and non-CNS tissues. Andersen et al. (2020) (29) combined cortical organoids with skeletal muscle spheroids to generate 3D cortico-motor assembloids. Importantly, optogenetic stimulation of cortical neurons in the model resulted in skeletal muscle contraction and thus validated its use in modelling diseases affecting the neuromuscular junction. Even though such an assembloid model has not been used yet in a disease modelling context, it holds significant promise for modelling disorders, such as spinal muscular atrophy (SMA) or amyotrophic lateral sclerosis (ALS). Cortical organoids can further be fused with vasculature elements to serve as an experimental model of infection. For instance, Wang et al. (2021), integrated pericyte-like cells (PLCs) into a cortical organoid model and the resulting structure could be infected by SARS-CoV-2. Moreover, the PLCs induced astrocyte maturation and mediated inflammatory type I interferon transcriptional responses, as demonstrated *in vivo*. Finally, assembloid models have been used to model interactions between the CNS and the intestine (Son et al., 2017). The growing interest in the field of brain-gut interactions has facilitated the development of such structures, which demonstrate significant potential for modelling complex pathologies such as Parkinson’s disease (PD) or IBD.

A pioneering study by Son et al. (2017) underscored the effectiveness of 3D multicellular cultures for comparing neural and intestinal components of PD. Here, cultures of 3D human neuroectoderm spheres and human intestinal organoids harboring the most common PD mutation, *LRRK2* G2019S, were generated from the same genetic patient background to identify molecular pathways affected by LRRK2 mutation. Altered expression of genes related to synaptic transmission was observed in both mutant cultures, revealing the first significant evidence of G2109S pathogenic role in intestinal cells. In conclusion, multicellular systems and assembloids have become in a very short time invaluable resources for understanding the pathophysiology and complexity of diseases affecting multiple systems, such as in PD.

## 4 Outlook and future perspectives

Organoids and assembloids are increasingly used to model the crosstalk between cell types in development and disease. Despite the clear potential of these 3D multicellular systems in modelling metabolic, intestinal, and neurological diseases, many limitations still exist, which need to be carefully considered. First, 3D multicellular systems generation can be time-consuming, complex, costly, and labor-intensive. In addition, important challenges remain in re-creating within multicellular systems the exact replicates of the *in vivo* tissue microenvironment, such as the tissue-tissue interface, spatiotemporal distribution of gases, nutrients and waste, and the mechanical microenvironment (Duval et al., 2017). In most of the examples so far available, 3D multicellular systems better recapitulated early developmental states instead of mature adult phenotypes and show high between-batch heterogeneity (de Jongh et al., 2022). This is due in part to the challenges of maintaining long-term cultures, with organoids forming necrotic cores due to a lack of nutrient permeability as they grow. Advances in culture techniques, such as the use of slice cultures, have begun to address these concerns (Humpel, 2015). Additionally, gaps in understanding *in vivo* organogenesis and tissue architectures lead to an incomplete or inaccurate generation of multicellular systems, affecting cellular diversity, specificity, maturation, and organization. Indeed, organogenesis relies on a complex interaction of multiple cell types from different lineages and vascularization, which often lack in organoids. Many groups have begun to address such a lack of cellular diversity using co-culture models (Takebe and Wells, 2019; Eicher et al., 2022; Kishimoto et al., 2022). Co-culturing cells from different lineages aims to better recapitulate cell interactions during tissue ontogeny. Moreover, co-culture models have been applied to areas where cell-cell interactions from different lineages are key to tissue homeostasis or in disease states, such as gut-immune (Noel et al., 2017), gut-brain (Workman et al., 2017; Park et al., 2020), gut-microbe (Puschhof et al., 2021) and neuro-immune (Abreu et al., 2018) interactions. Attention is also focused on engineering vascularized organoids to allow for improved nutrient diffusion and, therefore, prolonged culture and maturation through various techniques, such as co-culture with endothelial cells (Yin et al., 2016), bioengineering (Nashimoto et al., 2017) and grafting *in vivo* into the host tissue (Mansour et al., 2018). Thus, 3D multicellular systems require optimization, and the application of bioprinting (Murphy et al., 2020) to this field might present a solution to many of the challenges the field currently faces. Bioprinting is indeed an innovative technology that enables the assembly of multiple biological components (cells and biomaterials) in a precise ratio, integration of vasculature, high-throughput capability, and reproducibility (Murphy et al., 2020). Finally, the future generation of organoids or other forms of 3D multicellular models must achieve cellular complexity in a controlled manner and be inspired by organ development (Takebe and Wells, 2019). Wells and others provided a good example of this by generating multicellular gi organoids comprising cells from distinct germ layers (enteric neuroglial, mesenchymal, epithelial precursors) (Eicher et al., 2022). Notably, protocols to differentiate stem cells into different types of organ-specific supporting cells, either of mesodermal or ectodermal origin, have started to become available which has also leveraged signalling networks and molecular markers from single-cell transcriptomics (Kishimoto et al., 2022). The field now needs to define better strategies to combine these different cell types in appropriate ratios and spatial patterns, as *in vivo* in the tissues.

To conclude, we are now in a new age of 3D cellular cultures. The field is in constant expansion, benefiting from increasing collaborative and cross-disciplinary research. Whilst 3D multicellular systems have already proven to be a powerful tool for disease modelling, the ongoing concerted efforts will enable us to even more faithfully reproduce heterogeneous tissue constructs and move the field closer to clinical application.
